# Mechanical Durotactic Environment Enhances Specific Glioblastoma Cell Responses

**DOI:** 10.3390/cancers11050643

**Published:** 2019-05-09

**Authors:** Ilaria Elena Palamà, Stefania D’Amone, Patrizia Ratano, Amato Donatelli, Andrea Liscio, Giuseppe Antonacci, Mariangela Testini, Silvia Di Angelantonio, Davide Ragozzino, Barbara Cortese

**Affiliations:** 1 National Research Council-Nanotechnology Institute, 73100 Lecce, Italy; ilaria.palama@nanotec.cnr.it (I.E.P.); stefania.damone@nanotec.cnr.it (S.D.); mariangela.testini@nanotec.cnr.it (M.T.); 2 National Research Council-Nanotechnology Institute, 00185 Rome, Italy; patrizia.ratano@nanotec.cnr.it; 3 Department of Physiology and Pharmacology, Sapienza University, 00185 Rome, Italy; donatelli.1710684@studenti.uniroma1.it (A.D.); silvia.diangelantonio@uniroma1.it (S.D.A.); davide.ragozzino@uniroma1.it (D.R.); 4 National Research Council-Institute for Microelectronics and Microsystems, via del Fosso del Cavaliere 100, 00133, Roma, Italy; Andrea.Liscio@artov.imm.cnr.it; 5 Center for Life Nanoscience, Istituto Italiano di Tecnologia, 00185 Rome, Italy; giuseppe.antonacci@iit.it

**Keywords:** glioblastoma, cell movement, cellular microenvironment, mechanotaxis

## Abstract

*Background*: A hallmark of glioblastoma is represented by their ability to widely disperse throughout the brain parenchyma. The importance of developing new anti-migratory targets is critical to reduce recurrence and improve therapeutic efficacy. *Methods*: Polydimethylsiloxane substrates, either mechanically uniform or presenting durotactic cues, were fabricated to assess GBM cell morphological and dynamical response with and without pharmacological inhibition of NNMII contractility, of its upstream regulator ROCK and actin polymerization. *Results*: Glioma cells mechanotactic efficiency varied depending on the rigidity compliance of substrates. Morphologically, glioma cells on highly rigid and soft bulk substrates displayed bigger and elongated aggregates whereas on durotactic substrates the same cells were homogeneously dispersed with a less elongated morphology. The durotactic cues also induced a motility change, cell phenotype dependent, and with cells being more invasive on stiffer substrates. Pharmacological inhibition of myosin or ROCK revealed a rigidity-insensitivity, unlike inhibition of microfilament contraction and polymerization of F-actin, suggesting that alternative signalling is used to respond to durotactic cues. *Conclusions*: The presence of a distinct mechanical cue is an important factor in cell migration. Together, our results provide support for a durotactic role of glioma cells that acts through actomyosin contractility to regulate the aggressive properties of GBM cells.

## 1. Introduction

Glioblastoma (GBM) currently represents the most malignant and incurable form of brain tumours. A hallmark of GBM is its highly diffusive invasiveness of tumour cells into the surrounding brain which hinders efficacy of all existing treatments [1]. Glioma cells rarely metastasize to other tissues. However, more than often, single infiltrating cells are found throughout the brain parenchyma, dispersing rapidly along white matter tracts and blood vessel walls [2,3,4,5,6]. This infiltration precludes surgical removal and contributes to recurrence. Respect to their behaviour in the parenchyma, on blood vessel walls, glioma cells act and display different morphologies [7,8,9,10,11,12,13,14,15]. In the parenchyma, a multipolar morphology is usually observed [10,16,17], whereas, in blood vessels, glioma cells show a unipolar elongation. Moreover, glioma cells have shown to shift from a slow, random migration mode (exhibiting lower Rac1 and CDC42 activities), when crossing the parenchyma, to a fast migration course (exhibiting higher RhoA activity) on the linear blood vessels [15,18]. 

Differences in glioma behaviour respect to the parenchyma and the blood vessels are also related to variations in stiffness [19,20,21,22,23,24,25,26]. Mechanical cues within the extracellular matrix (ECM) environment play a major role in prompting and guiding GBM cells´ invasion. This process often is referred to as “durotaxis” or “mechanotaxis” [27,28,29]. Pilot work showed that effects of stiffness was cell type dependent, demonstrating that GBM cell migration of continuous glioma cell lines increased on more rigid substrates with actin stress fibre formation and focal adhesion maturation. Whereas primary patient-derived GBM lines showed a range of rigidity sensing phenotypes with some lines migrating faster on stiffer substrates, and others rigidity-independent [30,31,32]. Inconsistencies of cell responses to substrate stiffness reported in literature may also stem from the interference of other stimuli, such as topography and chemistry of the surface. Furthermore, the analysis of cells response to mechanical signals has been mainly conducted on mechanically uniform substrates, without the presence of mechanical cues which are, instead, experienced by glioma cells while dispersing within the brain. For this reason, studying cell migration in response to a mechanical cue would be important for understanding glioblastoma biology and glioma invasion during tumorigenesis, thereby providing an important tool to target more effectively the different stages of cancer progression. 

The mechanical cues of the microenvironment have shown to affect focal adhesions and actomyosin cytoskeletal arrangements through non-muscle myosin II (NMII) contraction and Rho-kinase [33,34,35]. Many glioma cell types grown on soft substrates exhibited reduced spreading, proliferation, presence of stress fibers, and focal adhesions respect to more rigid substrates [23]. An increase of stiffness involves an increased presence of integrin-associated structural and signalling proteins at the site of adhesion, as well as focal adhesion kinase (FAK), which in turn stimulates the Rho/ROCK (Rho kinase) pathway. Activation of ROCK promotes force generation and regulates myosin light chain (MLC) phosphorylation, NMII engagement with actin and contraction, leading to various cell processes, such as motility and adhesion [36]. Substrate stiffness affects also integrin clustering as well as focal adhesion assembly and turnover [37,38,39,40]. Actin filaments, besides, are implicated in the process of transducing signals by connecting to focal adhesion molecules such as integrins, vinculin and talin [41,42]. Studies suggest that a lack of actomyosin-based contractility reduces cytoskeletal tension in the spreading of cells on softer matrices [43,44]. Yet, it has also been shown that inhibition of non-muscle myosin II or ROCK in gliomas grown on soft substrates rescued their motility and adhesion to levels on compliant rigid substrates [23]. This underlies the fact that the understanding of tensional control of growth, adhesion and motility, gathered mostly from studies on rigid substrates, is incomplete.

In the present study we compare the rigidity dependent migration behaviour of U251 and GL15 GBM cell lines on uniform and durotactic polydimethylsiloxane (PDMS) substrates. In particular, these durotactic substrates present a double structure with underlying topographical features, which result in controlled mechanical cues projected on the overlying uniform membrane. Using long-term imaging, we found that the geometrical confinement induced by the mechanical cues plays an important and specific role in glioma migration and morphology, suggesting that the invasion of GBM can be conditioned also by the presence of a geometrical mechanical restriction.

We also examined the effect of the pharmacological inhibition of myosin activity with blebbistatin, Rho kinase with ROCK Y27632, or actin dynamics with cytochalasin D (Cyto. D) on cell response to different substrate rigidities. Results showed that inhibition of NMMII or ROCK corroborated a cell insensitivity to the mechanical environment, whereas inhibition of actin polymerization increased the mechanosensitivity.

This study provides the first evidence for GBM cell durotaxis on a definite mechanical cue and further defines stiffness gradient design parameters to support directed migration and cell elongation, questioning previous results reported on flat uniform rigid samples.

These findings can be important for the development of new therapeutic strategies which target invasiveness of GBM cells.

## 2. Results

### 2.1. Substrate Characterization

**Figure 1:**
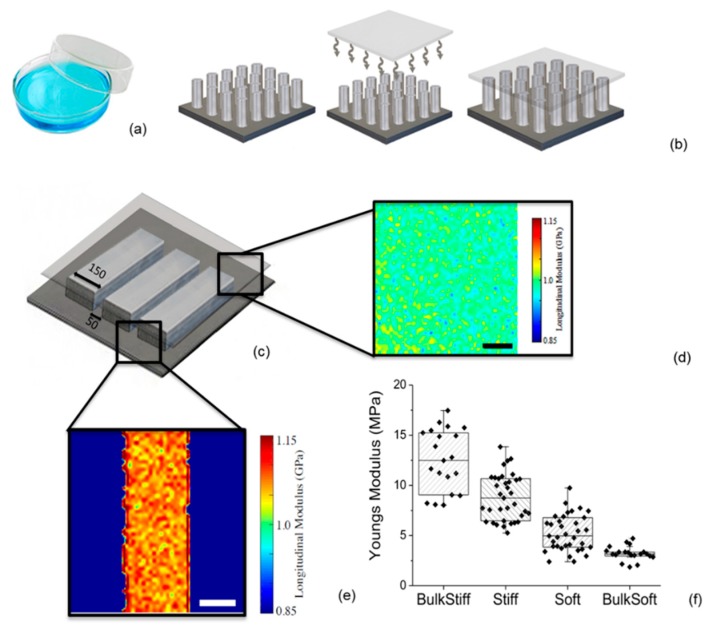
Design of complex mechanical PDMS substrates. (**a–b**) Schematic of substrates designed for this study. Bulk substrates were fabricated by simply pouring PDMS of the desired rigidity in petri dishes (**a**). For the durotactic substrates (**b**): a thin membrane of PDMS of controlled compliance is bonded on a topographically patterned stiff PDMS support, which generates stiffness cues at the substrate surface. The substrates used in this study included ‘line substrates’(**c**), and ‘flat substrates’. Brillouin microscopy was used to probe the surface longitudinal modulus, M′, for the PDMS (**d**,**e**). The thin membrane composed of 50:1 base to curing agent showed uniformity of measure with M′ = 0.988 ± 0.015 GPa (**d**); while the micropattern line of 150 µm displayed a longitudinal modulus of M′ = 1.070 ± 0.016 GPa, Scale bar: 100 µm. (**e**) Young’s elastic modulus of the different stiff and soft substrates assessed by AFM nanoindentation measurements (**f**). Statistical significance is *p* < 0.05, assessed by Tukey one-way ANOVA test.

To test the effect of matrix stiffness on cell migration and morphology, U251 and GL15 glioma cells were cultured on polydimethylsiloxane (PDMS) substrates. Bulk and micropatterned lines 50 × 150 µm and 15 × 15 µm were fabricated as previously described, and shown in Figure 1 [27,45].

Bulk uniform substrates were obtained by simply pouring a base to curing ratio of opportune rigidity (3:1 for very stiff and 50:1 with heptane for very soft) in petri dishes, as schematically shown in Supplementary Materials, Figure S1. We referred to these from here onwards as the bulk stiff and bulk soft substrates, respectively. 

Durotactic substrates with a stiffness cue were obtained by bonding a thin PDMS membrane, of base to curing ratio of 50:1 of about 10 µm, to the micropatterned substrate so as to display a topographically and chemically uniform substrate, while delivering a mechanical gradient dictated by the micropattern underlying the membrane. Generally speaking, a stiffer substrate was achieved with the PDMS membrane on the micropatterned lines (150 µm) while a softer substrate was obtained with the only thin membrane (without the underlying pattern, i.e., 50 µm) as schematically reported in Supplementary Materials, Figure S1. We referred to them from here onwards as the stiff and soft substrates, respectively. 

Quantification of the elasticity of these substrates were characterized in terms of nanoindentation and the longitudinal modulus. The longitudinal modulus of the thin membrane, measured with Brillouin microscopy, corroborated the presence of a uniform substrate without topographical variation which was estimated to be M′ = 0.988 ± 0.015 GPa, Figure 1d, lower than the underlying PDMS bulk substrate (M′ = 1.070 ± 0.016 GPa), Figure 1e. This confirmed that the PDMS lines can deliver a rigidity cue. Indentation arrays performed using a rigid spherical indenter AFM tip showed a Young modulus of the bulk stiff and bulk soft substrates respectively of E = 12.6 MPa and E = 3.2 MPa, and E = 9 MPa on the stiff substrate and 5 MPa on the soft, Figure 1f.

### 2.2. Glioblastoma Cell Morphology was Sensitive to Different Discrete Mechanical Stiffness in Particular to the Mechanically Uniform Durotactic Substrates 

**Figure 2:**
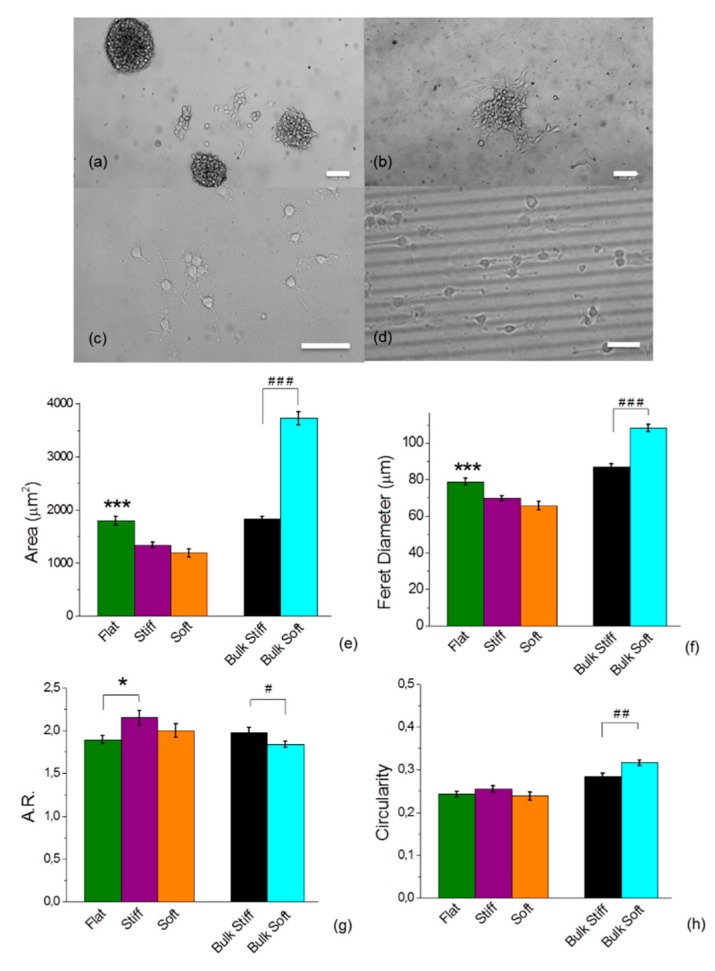
Substrate stiffness’s determines the distribution and morphology of the glioma cells. (**a**–**d**) Representative bright field images of U251 on bulk stiff (**a**), bulk soft (**b**), durotactic flat (**c**) and durotactic lined substrate (**d**) under 10× magnification (scale bars 100 μm). (**e**–**h**) Cell morphology analysis of area (**e**), Feret’s diameter (**f**), aspect ratio (A.R) (**g**) and circularity (**h**) were analysed with Fiji ImageJ. The value represents mean ± standard error (S.E.M) (*n* = 200 cells of 4 fields for each different condition). Statistical significance indicated by * for *p* < 0.05, ** for *p* < 0.01 and *** for *p* < 0.0001, assessed by Tukey one-way ANOVA test. The hash tag indicates statistical significance by two-tailed Student’s t-test analysis with # for *p* < 0.05, ## for *p* < 0.01 and ### for *p* <0.0001.

To delineate the effect of substrate stiffness on cell morphology we cultured both cell lines on the different mechanically uniform and micropatterned durotactic PDMS substrates. Both cell lines formed colonies and spherical aggregates when plated on the uniform bulk stiff and soft PDMS substrates but these were not observed on the durotactic substrates where cells were mostly consistently distributed. A higher number of smaller clusters in volume were observed on bulk soft substrates from which cells dispersed widely and more homogenously respect to the bulk stiff substrates where clusters were less and more voluminously grouped (Figure 2). 

These observations suggest that a lower stiffness of the ECM may interact more strongly with the cytoskeleton of cells from glioblastomas than that of higher stiffness. Quantitatively, cells cultured on the uniform bulk substrates showed a distinct morphologic phenotype as compared to those cultured on the durotactic substrates. In particular, on the different mechanically uniform substrates, we observed significant differences within the cell spread area, with a higher surface area on the bulk soft substrates for both cell lines (Figure 2 and Supplementary Materials, Figure S2). Whereas, the area on the mechanically gradient substrates was strongly reduced with the stiffness and geometrical mechanical confinement, although no significant differences were observed across the stiff and soft micropatterned substrates.

Shape descriptors such as the Feret diameter, the circularity ratio and axis ratio (A.R.) were also quantified. Large Feret diameters correspond to longer extensions from the cells, i.e., protrusions. A.R. basically represents a measure of how “elongated” is the cell’s shape. On the mechanically uniform bulk, U251 cells showed a lower A.R. on the bulk soft (Figure 2g) as opposed to the GL15 (Supplementary Materials, Figure S2g). This result reveals that GL15 were more elongated and produced more protrusions, whereas the U251 were larger and rounder. Cells of both lines were unexpectedly less elongated on the micropatterned durotactic substrates, (Figure 2f, Supplementary Materials, Figure S2f). 

The circularity represents how much the cell’s shape differs from a circle. A lower number for the circularity indicates a more stretched shape and/or longer/larger number of protrusions. In detail, this data reinforced the evidence that U251 on the bulk soft substrates were significantly rounder on mechanically uniform substrates, (Figure 2h). On the other hand, GL15 were more rounded on the stiffer substrates respect to the gradient durotactic substrates (Supplementary Materials, Figure S2h). 

**Figure 3:**
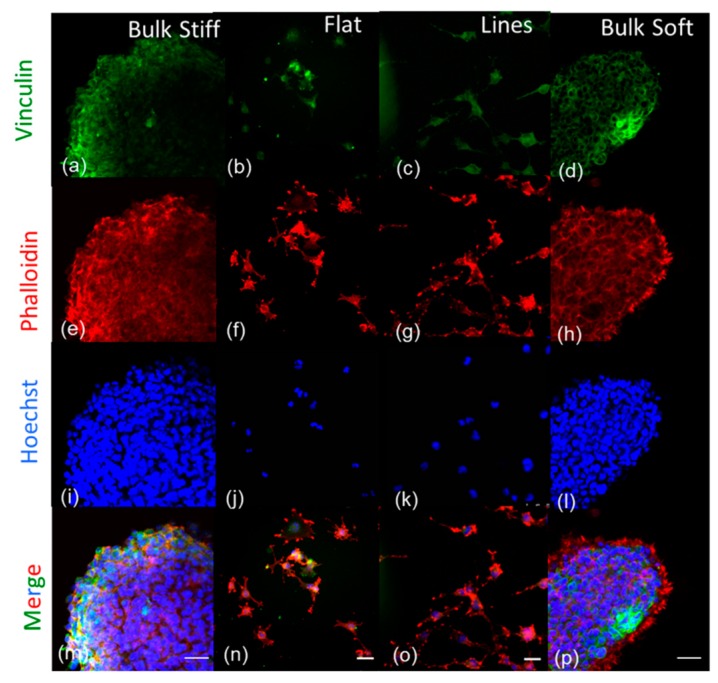
Distinctive mechanical stiffness in the diverse microenvironments is dependent on FAK signaling. Representative fluorescence images of phalloidin-stained F-actin, (**red**), vinculin for focal adhesion proteins (**green**) and HOECHST 33258 for cell nuclei (**blue**) of the U251 on the different durotactic substrates: bulk stiff (**a**,**e**,**i**,**m**), durotactic gradient flat (**b**,**f**,**j**,**n**), durotactic micropatterned substrate (Lines) (**c**,**g**,**k**,**o**) and bulk soft (**d**,**h**,**l**,**p**). The images are representative of one of three experiments conducted in duplicate. The images (**m**,**n**,**o**,**p**) are the merge of the (FITC, phalloidin-TRITC, and HOECHST 3325. Scale bars 100 µm.

We next explored the F-actin cortical cytoskeleton morphology and focal adhesions with phalloidin and anti-vinculin, respectively. Fluorescent images of U251 and GL15 on bulk and durotactic flat and micropatterned substrates are shown in Figure 3 and Supplementary Materials, Figure S3, respectively.

Both cell lines, on bulk soft substrates, displayed thin and short protrusions, with red fluorescence from phalloidin, representing F-actin fibres (Figure 3h, Supplementary Materials, Figure S3h) and a located clustering of adhesion components on the periphery of the cell (more evident for the U251 than the GL15). Cells on the bulk stiff substrates instead displayed focal adhesion and F-actin meshworks more delimited at the cell periphery, indicating that the stiffening of the surrounding area induces F-actin organization. Compared with the bulk substrates cells on durotactic substrates lost their rounded morphology and became stellate and protrusive with actin filaments aligned with the underlying pattern and focal adhesion complexes uniformly distributed.

### 2.3. Cell Speed is Differentially Regulated by Substrate Stiffness

**Figure 4:**
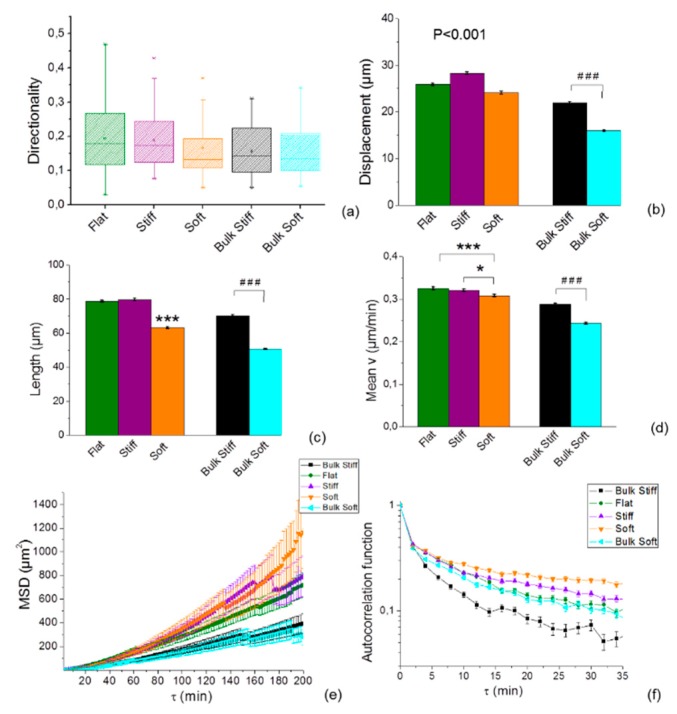
U251 glioma cells’ dynamic properties correlates to the mechanical stiffness of the substrate showing an increased response on the durotactic substrates. Plots show (**a**) directionality (**b**) net displacement, (**c**) path length, (**d**) average instantaneous speed of migration of cells imaged for over eight hours on the different substrates. (**e**) Plot of MSD vs. time indicating more directed motion for cells on the gradient durotactic micropatterned substrates than for cells on bulk substrates. (**f**) plot of average spatial autocorrelation function showing that on durotactic substrates U251 cell moves with a more highly correlated velocity than on the bulk substrates. Analyses were performed on tracks pooled from four independent experiments, from 40 cells. Statistical significance indicated by * for *p* < 0.05, ** for *p* < 0.01 and *** for *p* < 0.0001, were assessed by Tukey one-way ANOVA test. The hash tag indicates statistical significance by two-tailed Student’s t-test analysis with ### for *p* < 0.0001. Error bars indicate S.E.M.

To clarify which substrate was the most efficient to stimulate motility in both glioma cell lines, we investigated the speed dependency on the different substrate by performing time-lapse microscopy. Statistical analysis comparing directionality, total displacement, length and instantaneous speed on the uniform bulk and durotactic substrates showed a difference between cell lines (Figure 4). Both cell lines did not show a preferential directionality on the substrates (Figure 4a; Supplementary Materials, Figure S4a), but migrated further on stiffer substrates respect to the soft (Figure 4b,c, Supplementary Materials, Figure S4b,c). Comparison of instantaneous speed of the tracked cells on the stiff and soft durotactic substrates and on the bulk substrates also confirmed a higher migration rate in response to increasing substrate stiffness in both cell lines (Figure 4d; Supplementary Materials, Figure S4d). This indicated that stiffer substrates supported better migration of both glioma cell lines. 

The migration paths of the individual cells on the different substrates was further analysed through the mean squared displacement (MSD), which gives an indication on how far a cell migrates in a given time interval (Figure 4e, Supplementary Materials, Figure S4e). Interestingly, the MSD of the U251 cells on the durotactic substrate was greater than that on the bulk (see Figure 4e), consistent with the dynamic velocity data. On the other hand, the MSD of the GL15 cells monotonically increased with the bulk substrate stiffness respect to the durotactic substrates (Supplementary Materials, Figure S4e). Moreover, the MSD on the durotactic substrates was indistinguishable for stiff and soft durotactic rigidities, showing a rigidity insensitivity of both cell lines. Typically, in a diffusive process, MSD is a linear function of time, whereas a non-linear dependence in time of the MSD is found in anomalous diffusion processes. By plotting $\langle \Delta r^{2}\left(\tau \right)\rangle$ as a function of time interval, we obtained $\langle \Delta r^{2}\left(\tau \right)\rangle \propto t^{1.6}$ in U251 cells and $\langle \Delta r^{2}\left(\tau \right)\rangle \propto t^{1.4}$ in GL15 cells. These results indicate that (i) GL15 cells movement on all substrates are more or less super-diffusive, and that (ii) U251 cells movement are strongly super diffusive. This suggests that the U251 on the durotactic substrates have more active cellular migrating processes and cells spread more widely after long time periods.

Because both the directionality and the MSD are strongly biased by speed, we computed also the direction autocorrelation, which measures how angles describing the trajectory are aligned with each other. The direction autocorrelation plot confirmed a difference of migration of both cell lines on both bulk and durotactic substrates (see Figure 4f, Supplementary Materials, Figure S4f) [46].

The curve of U251 cells decays slower over time for durotactic substrates rather than on the bulk where they rapidly decreased to zero. This result indicates a higher degree of directionality on bulk substrates for U251 cells as opposing to the GL15 cells, confirming a strong effect of the mechanical geometrical gradient on cell movement.

### 2.4. Cell Viability is not Influenced by Substrate Stiffness

Cell growth and apoptosis are closely related to cell shape and adhesion, therefore the sensitivities of U251 and GL15 cells to the durotactic substrates were analysed by measuring cell viability with the MTT assay (Supplementary Materials, Figure S5) after 24, 48, 72 h after culture. Results indicated higher ratio of cell viability among both cell lines for cells cultured on durotactic flat substrates respect to bulk substrates. The highest viability decrease (20%) was observed for GL15 cells between the bulk stiff and soft substrates whereas the same was observed for the U251 only after 48 and 72 h. Lower viability decreases for U251 are consistent with their growth.

To quantitate the durotactic induced apoptotic cell death in U251 and GL15 cells, approximately 105 U251 and GL15 cells were double stained with Annexin-V-FITC and propidium iodide (PI) on the durotactic, bulk stiff and bulk soft substrates. FACS analysis identified less apoptic cells on the flat durotactic substrates.

### 2.5. Inhibition of Myosin and RhoA is Morfologically Affected by the Durotactic Substrate Stiffness but not Dynamically

**Figure 5:**
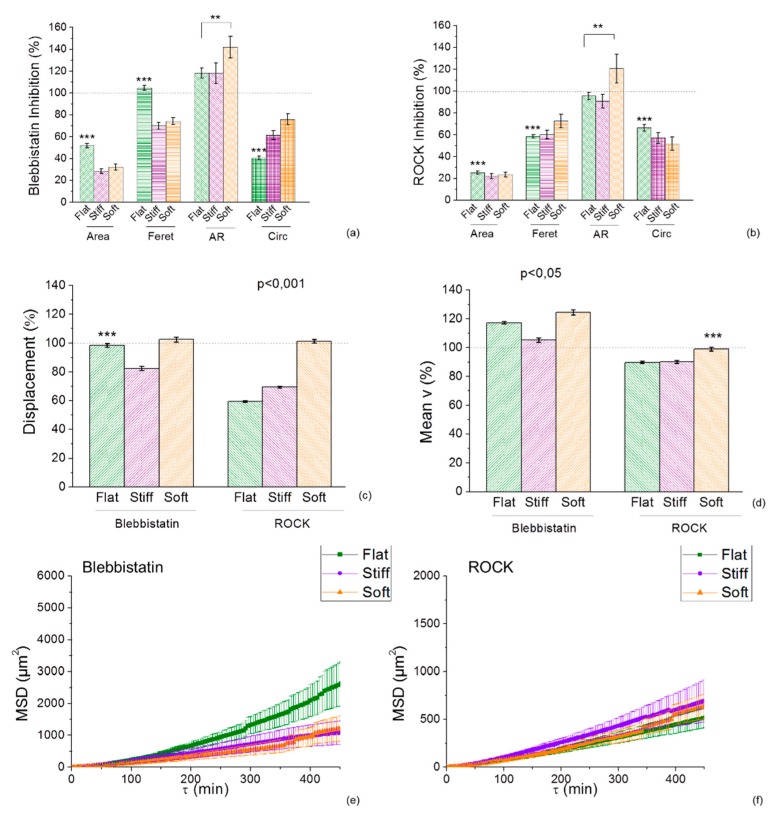
Durotactic substrates altered the responses of U251 cells to inhibition of NMMII and ROCK Y27632, responsible for cytoskeleton assembly and cell contractility. U251 cell morphology subsequent inhibition was sensitive to the durotactic micropatterned substrates compared to uniform substrates. Effects of blebbistatin (**a**) and ROCK Y27632 (**b**) on cellular spread area, elongation (Feret diameter), A.R. and Circularity for each substrate stiffness. Treated cells (bars) are compared with that in control cells (dotted lines). Relative to the morphologic cell response on uniform substrates, U251 decreased their spread area and elongation on the durotactic gradient substrates. Cell migration observed by time-lapse video microscopy was similar across all mechanically uniform substrates evaluated. Cell track positions were used to calculate the displacement (**c**), speed (**d**) of migration and the mean squared displacement (**e**,**f**) with blebbistatin (25 µM, 8 h), and ROCK Y27632 (10 µM, 8 h). Statistical significance indicated by * for *p* < 0.05, and *** for *p* < 0.0001, assessed by Tukey one-way ANOVA test. Error bars indicate S.E.M.

Collectively, our data suggests that both U251 and GL15 cell migration is influenced by the geometrical constrainments of the mechanical cues of the durotactic substrates. We therefore proceeded to determine whether RhoA/actin/cell rigidity signalling was involved behind the observed durotactic cell migration. In order to identify the cytoskeletal components contributing to cell motility observed in this study, we tested the role of inhibitors targeting the RhoA pathway including ROCK (Y-27632), the cytoskeleton-associated motor proteins components of myosin II (blebbistatin) (Figure 5, Supplementary Materials, Figure S6). 

In the presence of blebbistatin at a concentration of 25 µM (an inhibitor of non-muscle myosin II), and of the Rho-associated kinase inhibitor ROCK Y27632 at a concentration of 10 µM, both cell lines assumed a stellate-shape showing a lack of actin fibril organization. Qualitatively, cells treated with blebbistatin showed a reduced area and a less elongated morphology on the stiff and soft lines respect to the flat (Figure 5a, Supplementary Materials, Figure S6a). Similarly, inhibition of ROCK via Y27632 showed a decrease of the spread area but no significant differences between elongation, aspect ratio and circularity across uniform and the micropattern of durotactic substrates were observed (Figure 5b). The opposite was seen for the GL15 cells treated with blebbistatin which showed an increase of the area and elongation particularly on the micropatterned durotactic substrates (Supplementary Materials, Figure S6a). Inhibition of ROCK reduced the area of cells, which, however, were larger and more elongated on the durotactic micropatterned substrates respect to the flat (Supplementary Materials, Figure S6b).

Quantitatively, inhibition of blebbistatin tracks increased U251 speed especially on the soft micropatterned sections of the durotactic substrates (Figure 5c,d) whereas Y27632 reduced glioma cell migration on the flat and stiff micropatterned sections respect to the soft which remained quantitatively the same (Figure 5c,d). On the other hand, we found that blebbistatin reduced migration of GL15 cells on the micropatterned durotactic substrates respect to the flat (Supplementary Materials, Figure S6c,d), as well as inhibition with Y27632. 

Comparison of the MSD plots of migration of both cell lines inhibited with blebbistatin, indicated that migration was higher for the flat uniform substrates respect to the micropatterned durotactic ones (Figure 5e, Supplementary Materials, Figure S6e). This implicates that the mechanical cue induced a more confined movement of cells respect to when cultured on flat uniform substrates. Whereas, MSD analysis of ROCK inhibition via Y27632 of both cell lines presented a mean cell speed on the flat substrate, approximately equivalent to the durotactic substrate, suggesting that the ROCK-myosin II pathway was not implicated on the observed durotactic cell migration (Figure 5f, Supplementary Materials, Figure S6f). 

### 2.6. Inhibition of Actin Polymerization Increases Mechanotaxis of Cells

**Figure 6:**
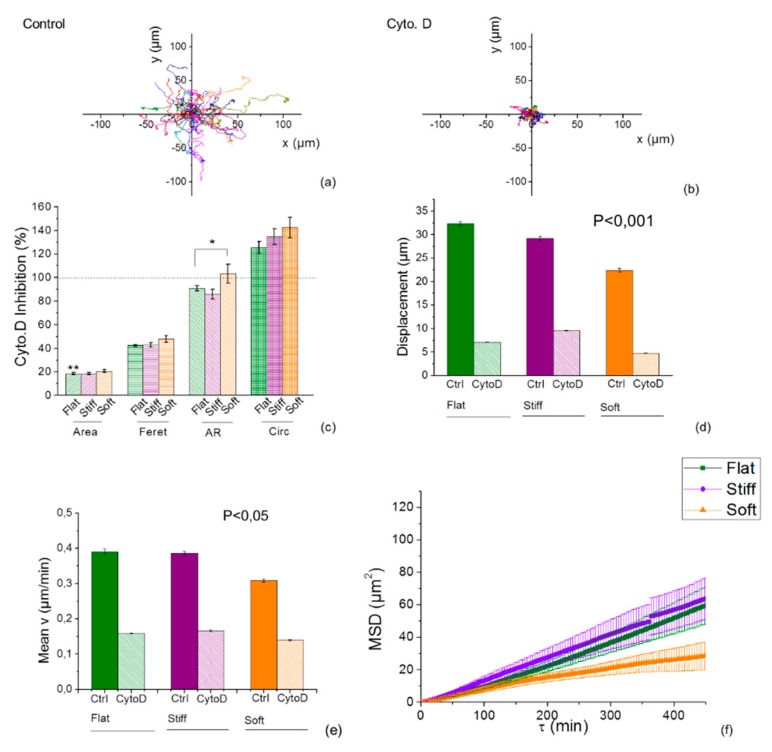
Inhibition of actin polymerization with Cyto. D on U251 on durotactic substrates showed a stiffness-dependent reduced response with a higher sensitivity to stiff substrates. (**a**,**b**) Cell movement track of 35 cells from 4 fields of acquisition on flat and micropatterned durotactic substrates was analysed by ImageJ. The different colours represent different cells analysed. (**c**) Normalized U251 cell morphology such as cellular spread area, elongation (Feret diameter), A.R. and Circularity of cells subsequent treatment with Cyto. D, where treated cells (bars) are compared with that in control cells (dotted lines). Cell track positions were used to calculate the displacement (**d**), speed of migration (**e**) and the mean squared displacement (MSD) (**f**) as a function of the time lag (τ) of U251 cells treated with Cyto. D. Statistical significance indicated by * for *p* < 0.05, assessed by Tukey one-way ANOVA test. Error bars indicate S.E.M.

To investigate the involvement of the actin cytoskeleton, both cell lines were treated with Cyto. D, an actin-disrupting agent, at a concentration of 1 µM, Figure 6, Supplementary Materials, Figure S7). 

Comparison of wind-rose plots of tracked cells indicated that migration decreased in response to inhibition with Cyto.D (Figure 6a,b; Supplementary Materials, Figure S7a,b). The inhibitor interfered also with the cellular spreading (Figure 6c, Supplementary Materials, Figure S7c) preventing protrusion formation. Imaging with time-lapse microscopy, showed suppressed actin assembly of U251 and GL15 cells with impaired motility and lack of movement, but the effect of Cyto. D on the sensitivity to the mechanical cue of the substrate appeared to be cell specific. The average speed and the overall migration were dramatically reduced in both cell lines upon Cyto. D treatment (U251 median speed was reduced of 43.1% on the flat respect to its control, and about 42 ± 3% on the stiff and soft lines, Figure 6e; GL15 median speed reduced of 35.7% in control, and 29.2 ± 0.4% on the stiff and soft lines Supplementary Materials, Figure S7e), indicating that cells are dependent on actin filaments. MSD vs. time curves showed a confined motility for both cell lines but also that substrate stiffness effected cell motion and this effect is stronger for stiffer substrates with U251 faster on stiff lines (Figure 6f) as opposed to GL15 cells (Supplementary Materials, Figure S7f). 

Taken together this evidence points to a role for a binding protein or a receptor-mediated mechanism in connection, to the substrate stiffness, indicating that actin was involved, although U251 cells seemed to be more sensitive to actin depolymerization than GL15 cells.

### 2.7. Effects of Inhibition on F- actin and Focal Adhesions Organization on GBM Cells

Cell adhesion response to the different substrates could result from the different expression of cellular adhesion molecules [43,47,48]. Activation of Src kinase and FAK have been shown to promote cell migration. FAK is activated by autophosphorylation of Tyr397, a site recognized by Src kinase. Src regulates cellular migration through substrates. For example, Src phosphorylates and modulates focal adhesion assembly and migration [49]. Our results on Fak and p-FAK Tyr397 agree with previous reports on substrates with varying degrees of stiffness showing that phosphorylation levels of U251 cells increased with increasing substrate rigidity. To establish that blebbistatin, Y27632 and Cyto. D were in fact inhibiting Rho kinase and decreasing FAK phosphorylation, we quantified the changes in FAK and FAK phosphorylation. 

**Figure 7:**
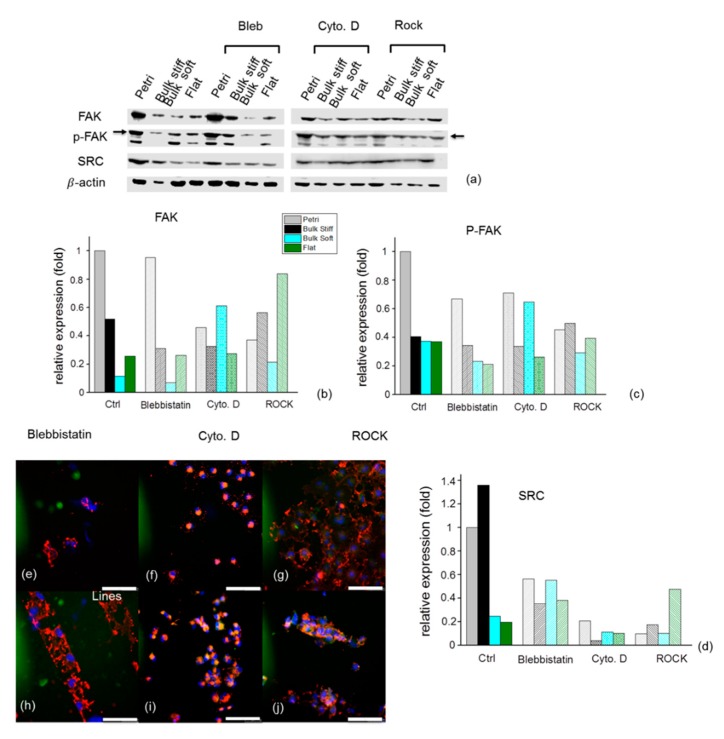
FAK, Phosphorylation of FAK Tyr397 and SRC in glioma cells depends on substrate stiffness. Western blot analysis (**a**) and densitometry of (**b**) FAK, (**c**) phosphorylation of FAK Tyr397 and (**d**) SRC levels in lysates from U251 cells cultured on bulk stiff, bulk soft and flat durotactic, the Ctrl is the Petri dish. Quantified values hown in the graph (*n* ≥ 3). FAK, P-FAK and Src expression levels were normalized to the β-actin. Immunofluorescence staining merge of phalloidin, vinculin and HOESCHT showing actin cytoskeleton organization of U251 cells treated with inhibitors on flat substrates with (**e**) blebbistatin (**f**) ROCK Y-27632, and (**g**) Cyto D and micropatterned durotactic lines with (**h**) blebbistatin (**i**) ROCK Y-27632, and (**j**) Cyto. D. Scale bar 100 µm.

Western blot analysis confirmed the expression of FAK and phosphorylated FAK on bulk stiff, bulk soft and flat durotactic substrates indicated by the bands (see Figure 7, Supplementary Materials, Figure S8) corresponding to these kinases. By inhibition of actomyosin contraction with blebbistatin, FAK and phosphorylation levels were slightly reduced on the bulk stiff and bulk soft substrates. Remarkably with Cyto. D treatment these levels increased predominantly on the bulk soft substrates. On the contrary for the GL15, inhibition of blebbistatin and ROCK increased FAK phosphorylation on the stiff bulk substrates whereas these were lower for Cyto. D. These results confirm that FAK is a candidate for the adhesion signalling pathway, which exhibits a site-specific response to substrate stiffness. Involvement of Src signaling in the control of cell migration also confirmed FAK expression.

Confocal images of U251 and GL15 cells pre-treated with blebbistatin, Y27632 and Cyto. D cultured either on flat compliant substrates or the durotactic substrate (stiff and soft lines) revealed disruption of stress fibres and clear decrease of the bundling of actin stress fibres and the relative size of the focal adhesions in focal adhesion after treatment (Figure 7e–j, Supplementary Materials, Figure S8e–j). Cells of both lines treated with Cyto. D exhibited disrupted actin fibril organization. 

## 3. Discussion

Cell behaviour is strongly influenced by the biophysical cues of the environment. Previous studies established that cells behave differently on substrates of distinctive uniform rigidities [23,31,44,50]. Our work extends this current body of knowledge, providing evidence that the presence of mechanical cues of the underlying surface can differentially modulate glioma cell behavior respect to uniform substrates. Specifically, the presence of a mechanical cue regulated, in a cell line specific manner, the morphology, the anchorage and the motility of glioma cells. This work is the first study to our knowledge which shows discrimination of these parameters based on the presence of a mechanical cue.

In particular, results showed that glioma cells adhered differently to the various substrates. Remarkably, we noticed that both U251 and GL15 cells formed initially aggregates only on stiff and soft bulk substrates (Figure 2). Of note, it appeared that on stiff bulk substrates there was a reduced amount of aggregates, with promotion of cell–cell cohesion at the advancing cell front, where cells were tightly adherent to one another (Figure 3). Whereas on the bulk soft substrates, a higher number of aggregates was observed with cells dispersing from the aggregates as single cells from the advancing edge, with shorter protrusions spreading from the cells (Figure 3). This suggests that cells may actively reorganize their actin cytoskeleton to probe and invade the matrix. Conversely, on the durotactic substrates cells were more uniformly spread, deprived of aggregates with a more spindle-like morphology.

Previous reported studies suggest that the different rigidity-dependent responses might reflect sub-class specific behaviours of cell lineages [31,32]. For example, Grundy and coworkers (2016), reported rigidity-dependent responses between patient derived cell lines. Some cultures spread and generated actin stress fibres on more rigid substrates, while others showed ridigity-insensitive phenotypes [32]. Consistently, we observed cells more well spread on the durotactic substrates respect to uniform substrates. However, the rigidity and geometrical mechanical confinement of the substrate induced a smaller size and a more elongated shape of cells. 

The durotactic cues also strongly influenced cell migration response respect to the bulk uniform substrates. It has been reported that substrate rigidity can induce different migratory responses in different tumour cells [32]. In our study, the two investigated cell lines showed significant similarities, as both exhibited an increased speed of migration on the stiff substrates. However, cells also exhibited distinct behaviours, with U251 showing an increase of cell speed on the durotactic cues respect to the bulk while the GL15’s displayed an opposite behaviour (Figure 4d, Supplementary Materials, Figure S4d). Cell migration may occur through different modes of migration which can be mesenchymal or amoeboid depending whether cells generate paths in the matrix via secretion of matrix metalloproteinases (MMPs), or employ morphological alterations to move through pores in the matrix, respectively [51]. The aspect ratio can be used to distinguish between mesenchymal and amoeboid phenotypes [52]. Cells with ratio of the major to minor axes higher than 1.5 are ranked as mesenchymal, whereas a value below 1.5 indicates an amoeboid phenotype. Based on the value of the aspect ratio, both U251and GL15 showed a mesenchymal phenotype (Figure 2g; Supplementary Materials, Figure S2g), confirming a stiffness dependent motility.

Several studies have described contradicting roles of the Rho GTPases and myosin II in GBM tumor progression, due to the different experimental systems reported [23]. For example, responses in glioma invasion with suppression of myosin II and/or activation of Rac and RhoA are significantly variable depending on physical microenvironments. Indeed, continuous culture models of GBM lines have shown to necessitate of myosin II and its upstream regulators to sense and respond to ECM rigidity [23,44,53], while primary cell lines were reported to be rigidity-insensitive [31,32]. Interestingly, treatment with either Y27632 or blebbistatin supported the notion of cell lines insensitivity to a rigidity gradient on our substrates. This would suggest that these treatments do not exert a significant selective effect.

An increase of the RhoA activity in glioblastoma cells has been associated with decreased motility and invasiveness and formation of stress fibres and focal adhesions [54,55]. Moreover, we observed in all substrates a reduced expression of vinculin (Figure 7, Supplementary Materials, Figure S8) in the presence of blebbistatin or Y27632. This was consistent with several studies, showing that inhibition of myosin II contractility or Rho kinases affects the process of integration of key mechanosensing proteins, such as vinculin, into focal adhesions [35,56].

Earlier evidence reported that actin cytoskeleton perturbation with cytochalasin D reduced cell movement, regardless substrate stiffness [23,57]. We, too, observed that inhibition of actin polymerization interfered with cell motility. Moreover, we observed that cytochalasin D treatment restored sensitivity to the ECM stiffness. However, U251 were faster on stiff substrates while GL15 were slower.

Also, we did not observe vinculin positive focal adhesions, while cells reverted to a rounder shape. This suggested that cytochalasin D led to adhesions failure rather than maturation. Disruption of actin filaments has showed to induce proMMP-2 activation and loss of MMP-9 expression [58,59,60]. Our preliminary efforts to identify the pathways that control the stiffness dependency did not point out obvious candidates, indicating that more work is needed to elucidate the underlying molecular network controlling this cellular response. 

In summary, we have documented an intriguing role for durotactic cues respect to uniform substrates in regulating the balance between adhesion and motility of different cell lines. Generally, studies in literature of migration on 2D surfaces do not adequately represent the mechanical constraints present within the brain. Using PDMS elastomers, which allowed to modulate the rigidity of the substrate, we demonstrated that the presence mechanical cues can induce different changes in glioma cells. This kind of substrate can provide additional information respect to normally used rigid uniform surfaces. Indeed, using a durotactic substrate, we show that interfering with actin-polymerization dynamics, effectively blocked cell migration. In addition, we also provide evidence that this treatment highlights a cell dependent sensitivity to the substrate rigidity. 

The results reported in this study may facilitate the development of optimal in vitro platforms to mimic in vivo conditions to study cancer cell migration and to uncover therapeutic strategies against tumour cell motility and invasion.

## 4. Materials and Methods

### 4.1. Materials

All tissue culture media were purchased from Life Technologies (Carlsbad, NM, USA). All chemical reagents were obtained from Sigma-Aldrich (Steinheim, Germany), unless otherwise stated. AnnexinV-PI kit was acquired from Abcam (Cambridge, UK), Coomassie brilliant blue staining from BioRad (Hercules, CA, USA).

### 4.2. Cell Cultures

Glioblastoma-derived human cell lines U251and GL15 (kindly provided by Dr. Emilia Castigli, Perugia University) were cultured in DMEM (Invitrogen, San Diego, CA, USA) supplemented with 10% heat-inactivated FBS (Invitrogen), 100 IU/mL penicillin G, 100 μg/mL streptomycin, 2 mM glutamine, and 1 mM sodium pyruvate. Cells were grown at 37 °C in a 5% CO_2_ humidified atmosphere.

### 4.3. Preparation of PDMS Uniform and Gradient Durotactic Substrates

To obtain chemically and topographically uniform substrates of varying stiffness gradients, double sandwich polydimethylsiloxane substrates were used. Substrates were made according to previous reports [27]. Substrates were washed and sterilized with 70% ethanol. To promote cell adhesion, PDMS surfaces were treated with 100 μg/mL poly-l-lysine 1 mg/mL under UV light for ~15 min. 

### 4.4. Characterization of Substrates

#### 4.4.1. Brillouin Measurements

A custom-built Brillouin microscope was used to measure the spatial distribution of the membrane stiffness and underlying pattern. Brillouin microscopy with a single-longitudinal mode laser beam (Verdi V12, Coherent, Santa Clara, CA, USA) was applied and focused on the membrane using a high numerical aperture (NA = 1.4) objective lens. The light scattered inelastically by the sample was collected in confocal mode using a single mode optical fiber (460P, Thorlabs, Munich, Germany) and analysed through an apodized single-stage VIPA spectrometer. [61] From the measured Brillouin frequency shift (νB) of the Brillouin peaks, the real part of the longitudinal modulus (M′) indicative of the material stiffness was estimated using the relationship M′ = ρ(λνB/2n)^2^, where ρ = 965 kg/m^3^ is the material density, n = 1.4 the refractive index and λ = 532 nm the laser wavelength.

#### 4.4.2. AFM Measurements

Mechanical characterization was performed at nano- and micro- scale by atomic force microscopy (AFM). Measurements were performed in air by employing a commercial digital microscope Multimode 8 (Bruker, Milan, Italy) using two different probes: a silicon nitride SPM with 8 nm apical radius (RFESPA, Bruker) and 6.6 um radius colloidal particle mounted on silicon nitride cantilever (CP-PNPL-SiO-C, sQUBE). Preliminary topographic maps were performed acquiring tapping-mode AFM images with RFESPA probes. The tip was indented into the PDMS substrate at a rate of 2.5 μm/s to produce a force-distance curve. Experiments were conducted across multiple samples prepared independently, with 20 measurements within a 100 × 100 μm^2^ area, which was averaged to give each independent value to be used in data representation and statistical analysis. Young’s modulus, or stiffness of the substrate was calculated from the force curves according to Hertz model which describes the elastic deformation (E).

### 4.5. Cell Morphological Analysis

Cells of related durotactic and inhibition experiments were fixed for 20 min with 4% (*w/v*) paraformaldehyde in phosphate buffer saline (PBS), and stained with 0.1% Coomassie Brilliant Blue in 50% methanol, 10% acetic acid for 1 hr and destained with 10% methanol and 7.5% acetic acid. Substrates were subsequently visualized using an inverted microscope (Olympus) equipped with a QImaging (Crisel, Rome, Italy) camera with 10× objectives (Plan N, NA = 0.25, Ph1) or 20× (LUCPlan FLN, NA = 0.45, Ph2). Brightfield images of 10 random microscopic fields were acquired per sample. Cell morphology was characterized using the particle measurement feature within ImageJ (www.nih.gov) to obtain spread area, circularity, aspect ratio (A.R.) and Feret’s diameter of single cells. Circularity of cells were obtained using the formula: Circularity = 4π (area/perimeter^2^). A circularity value of 1.0 indicates a perfect circle, and values near zero indicate a more elongated morphology of cells. Feret’s diameter is a measure of cell length, and represents the highest distance between any two points along the cell perimeter. The aspect ratio (A.R) is defined as the ratio between the major axis (M) and the minor axis (m) of the cell’s fitted ellipse, calculated by the ImageJ software.

### 4.6. Time-lapse Microscopy and Quantitative Analysis of Cell Migration 

Cells were seeded on the bulk and durotactic PDMS substrates 24 h before imaging. Time-lapse imaging was conducted on an Olympus IX73 inverted microscope, equipped with a QImaging OptiMOS sCMOS camera (QImaging, Surrey, BC, Canada) and in a stage-mounted incubator with CO_2_ and temperature control (H201; Okolab, Pozzuoli, Italy). Bright field images were acquired every 2 min using a 10× (Plan N, NA = 0.25, Ph1) or 20× (LUCPlan FLN, NA = 0.45, Ph2) objective. Acquisitions were typically acquired over a period varying from 8 to 10 h. Cell bodies were tracked using the manual tracking plugin (mtrackj) for Fiji software over 8 h of migration as previously described [62]. Individual cell tracks from time-lapse microscopy were recorded over time, and set to a common origin for spatial comparison. Results from a minimum of three separate experiments with up to 30–40 individual tracked cells were pooled for data analysis. The trajectories and parameters such as track lengths, displacement (Euclidean distance at each time point), cell instantaneous speed, directionality (track length [the last position minus the initial position] divided by the total displacement of the cell), mean square displacement (MSD) and direction autocorrelation function were plotted using Origin software. The MSD, and direction autocorrelation were calculated using DiPer software. [46] MSD was computed for the tracked cells within each experimental condition and calculated as follows:

< *MSD*(τ)) > = < (*xi*(t + τ)−*xi*(*t*))^2^ + (*yi*(t + τ)−*yi*(*t*))^2^ >,

where *xi* and *yi* denote position of the *i*th cell in an experimental timelapse frame (*x*, *y*), and τ represents the time lag interval. The slope of the linear portion of the MSD curve was fitted in order to characterise the dynamic motion for each of the cells by the following equation [63]:

< *MSD*(*t*) > ∝ τ *α*

which allowed the power exponent α of the MSD curves to be calculated. If α < 1, a sub-diffusive population is observed, whereas if 1 < α < 2 a super-diffusive is denoted [46]. A one-way Anova test for the mean was used to assess the difference between the mean cellular values across experimental conditions.

### 4.7. Cell Viability Assay

In vitro cytotoxicity of different substrates against U251, and GL15 cells (5000 cells/mL) was evaluated via MTT assay for 24, 48 and 72 h of incubation, according to the manufacturer’s instructions (Sigma-Aldrich, Milan, Italy). 

The absorbance was spectrophotometrically measured at a wavelength of 570 nm and the background absorbance measured at 690 nm was subtracted. The percentage viability was expressed as the relative growth rate (RGR) by following equation:

(1) $$\mathrm{RGR}\left(\%\right)=\frac{D_{\mathrm{sample}}}{D_{\mathrm{control}}}\times 100$$

where D*_sample_* and D*_control_* were the absorbance of the sample and the negative control. Each experiment was repeated three times in triplicate (Student’s *t*-test, *p* < 0.05).

### 4.8. Apoptosis Analysis

Cell apoptosis was analyzed by flow cytometry. Briefly, 105 U251 and GL15 cells were seeded of different substrates for 24 h at 37 °C, 5% CO_2_. After incubation, U251 and GL15 cells were washed with PBS 1× and staining with Annexin V-FITC/PI according to the manufacturer’s instructions (Abcam). Cell apoptosis and cell cycle distribution were determined by analyzing 10,000 ungated cells using a Flow Cytometer (C6, Accuri, Milan, Italy). All experiments were performed in triplicate (Student’s *t*-test, *p* < 0.05).

### 4.9. Inhibition of Cell Contractility

NMMII inhibitor blebbistatin (Sigma-Aldrich), rho-associated kinase (ROCK) inhibitor Y-27632 (Sigma-Aldrich), and actin polymerization inhibitor cytochalasin D (Sigma-Aldrich) were added to the cell culture media in related timelapse and immunofluorescence experiments after the cells had been allowed to adhere for at least 24 h.

### 4.10. Western Blotting Analysis

Cell protein extract, from GL15 and U251, were obtained using RIPA lysis buffer containing the proteinase inhibitor cocktail. Samples were centrifuged at 10,000 rpm and the surnatant was collected. 30 μg of proteins were dissolved in sodium dodecyl sulfate (SDS) sample buffer and separated on 10% (*w/v*) polyacrylamide SDS gels. Separated proteins were transferred electrophoretically onto nitrocellulose membrane (Amersham Hybond ECL Nitrocellulose Membrane-GE, Abcam). The filter was blocked with 5% (*w/v*) non-fat dried milk in buffered saline. Blots were incubated overnight with specific primary antibodies directed against FAK 1:1000 (ZF002, Invitrogen), p-FAK 1:1000 (700255, Invitrogen), SRC 1:1000 (WH0006714M1, Sigma), β-actin 1:5000 (A1978, Sigma). The immune complexes were detected using peroxidase-conjugated secondary antibodies by chemiluminescence (Clarity™ Western ECL Substrate, BioRad). Densitometric analysis was carried out on the western-blots using the C-DiGit Blot scanner (LI-COR, Cornaredo Milano, Italy), normalizing to β-actin used as an internal control. 

### 4.11. Immunofluorescence

Cells were fixed using 4% paraformaldehyde in PBS for 20 min at room temperature before being permeabilized with 0.2% (*v/v*) Triton X-100 in PBS for 5 min and blocked with PBS containing 1% BSA. Cells were then labeled with primary antibodies anti-vinculin (mouse) antibody (Sigma) (1:100) and phalloidin-TRITC (Sigma) (1:500) in blocking buffer at 4 °C at 37 °C, and washed in PBS. Fluorescent dye (DYE-Light)-conjugated secondary antibodies against goat IgG were used at a dilution of 1:500 for 1 h at 37 °C in blocking buffer. After washing in PBS the samples were mounted with HOECHST 33258 (Sigma), 1 mg/mL in PBS 1× for 5 min. Cells were viewed on a Confocal microscopy system (Olympus) equipped with a 20× (UPlan FLN, NA 0.50), 40× (UPlanFLN, NA 1.30, oil) and 60× (UPlanSApo, NA 1.35, oil) with a resolution of 1024 × 1024 pixels.

### 4.12. Statistics

All statistical analysis was performed by using Origin 8 (Arezzo, Italy). Data was reported as mean  ±  standard error of the mean (S.E.M), a one-way ANOVA with Tukey’s post-hoc analysis was performed to determine differences amongst substrates. Data were considered statistically significant for a * *p* ≤ 0.05, ** *p* ≤ 0.01, and *** *p* ≤ 0.001.

## 5. Conclusions

In conclusion this study assesses glioma migration in a two-dimensional, topographically uniform surface comparing uniform surfaces with substrates with durotacic cues showing that the latter induces different morphological and dynamical response respect to simple uniform substrates. 

To our knowledge, this is the first study to report on the effects of a durotactic cue on glioblastoma cells. The results of our study demonstrate that tuning of durotactic stimuli can profoundly affect cell morphology and motility A better understanding of studies on extracellular matrix and cytoskeleton organization and the associated changes in gliomas will be of crucial importance due to the highly infiltrative nature of these tumours allowing to design treatments in the future.

## Supplementary Materials

The following are available online at https://www.mdpi.com/2072-6694/11/5/643/s1, Figure S1: Schematization of the substrates used to culture cells with different mechanical rigidities and cues. Figure S2: GL15 glioma cells’ dynamic properties correlates to the mechanical stiffness of the substrate showing an increased response on the durotactic substrates, Figure S3: Distinctive mechanical stiffness in the diverse microenvironments is dependent on FAK signalling, Figure S4: GL15 glioma cells’ dynamic properties correlates to the mechanical stiffness of the substrate showing a decreased response on the durotactic substrates, Figure S5: Durotactic flat substrates promotes glioma cell proliferation respect to uniform bulk, Figure S6: Durotactic substrates altered the responses of GL15 cells to inhibition of NMMII and ROCK Y27632, responsible for cytoskeleton assembly and cell contractility, Figure S7: Inhibition of actin polymerization with Cyto. D on GL15 on durotactic substrates showed a stiffness-dependent reduced response with a higher sensitivity to soft substrates, Figure S8: FAK, Phosphorylation of FAK Tyr397 and SRC in glioma cells depends on substrate stiffness.
