# Supplementary material for: Mechanical Durotactic Environment Enhances Specific Glioblastoma Cell Responses
Source: Cancers (Basel). 2019 May 9;11(5):643. doi: 10.3390/cancers11050643 (PMC6562761; doi:10.3390/cancers11050643)
Supplement: Supplementary file 1 [file cancers-11-00643-s001.pdf]

Supporting Information

Mechanical durotactic environment enhances specific glioblastoma cell responses

*Ilaria Elena Palamà[1], Stefania D'Amone[1], Patrizia Ratano[2], Amato Donatelli[3], Andrea Liscio[4], Giuseppe Antonacci[5], Mariangela Testini[1], Silvia Di Angelantonio[3,5], Davide Ragozzino[3], Barbara Cortese[*2]*

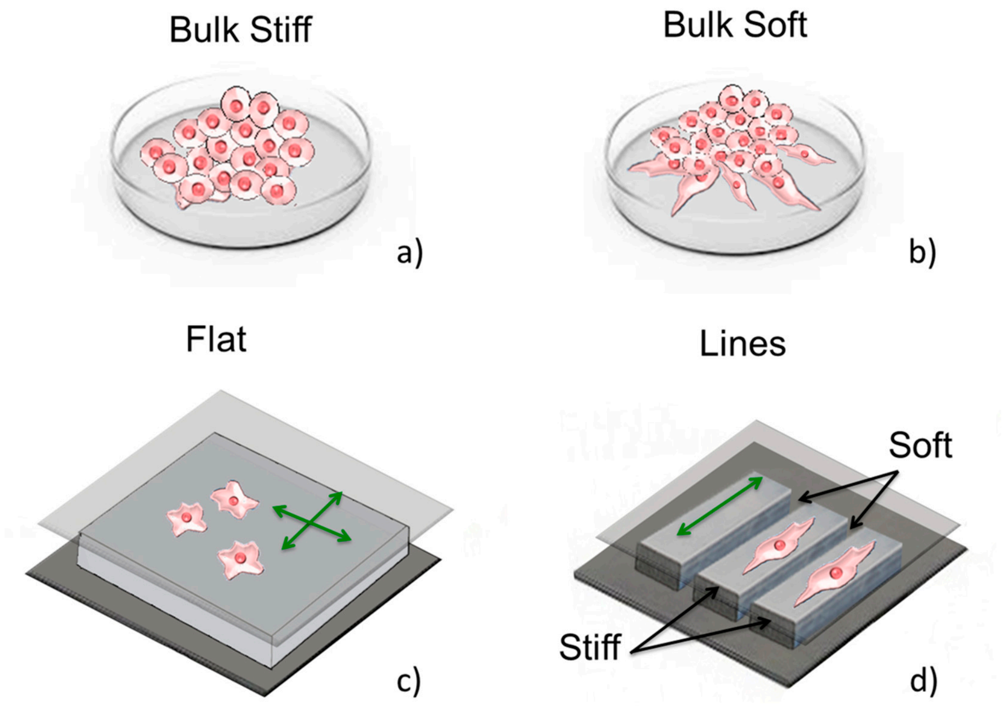

Figure S1: Schematization of the substrates used to culture cells with different mechanical rigidities and cues. (a) bulk stiff substrate, (b) bulk soft substrate, (c) flat durotactic substrates, (d) durotactic micropatterned substrates displaying the stiff and soft lines.

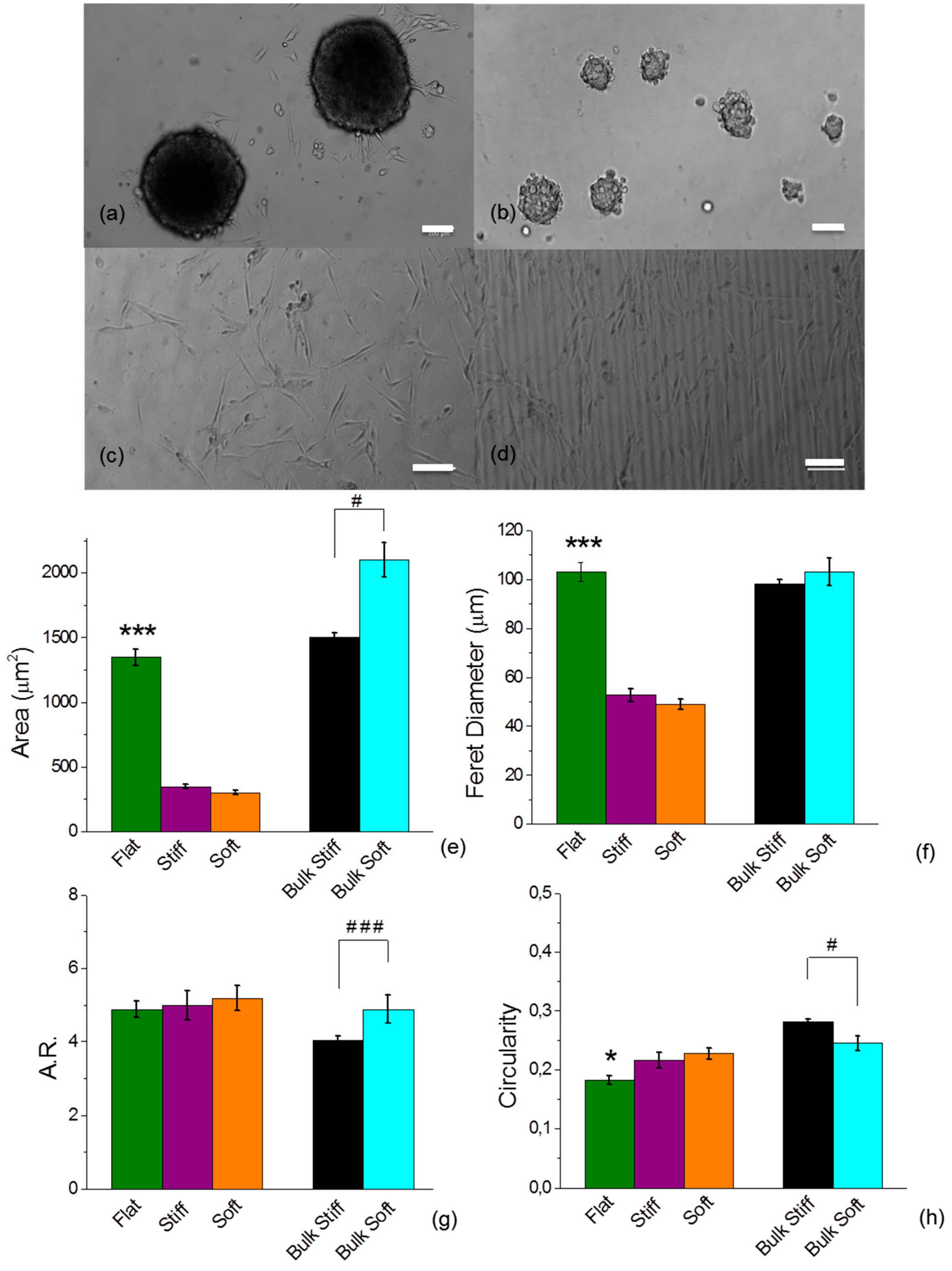

Figure S2: GL15 glioma cells' dynamic properties correlates to the mechanical stiffness of the substrate showing an increased response on the durotactic substrates. Plots show a) directionality b) net displacement, c) path length, d) average instantaneous speed of migration of cells imaged for over eight hours on the different substrates. e) Plot of MSD vs. time indicating more directed motion for cells on the gradient durotactic micropatterned substrates than for cells on bulk substrates. f) plot of average spatial autocorrelation function showing that on bulk substrates GL15 cell moves with a more highly correlated velocity than on the durotactic substrates. Analyses were performed on tracks

pooled from four independent experiments, from 40 cells. Statistical significance indicated by * for $p < 0.05$, ** for $p < 0.01$ and *** for $p < 0.0001$, were assessed by Tukey one-way ANOVA test. The hash tag indicates statistical significance by two-tailed Student's t-test analysis with # for $p < 0.05$ and ### for $p < 0.0001$. Error bars indicate s.e.m.

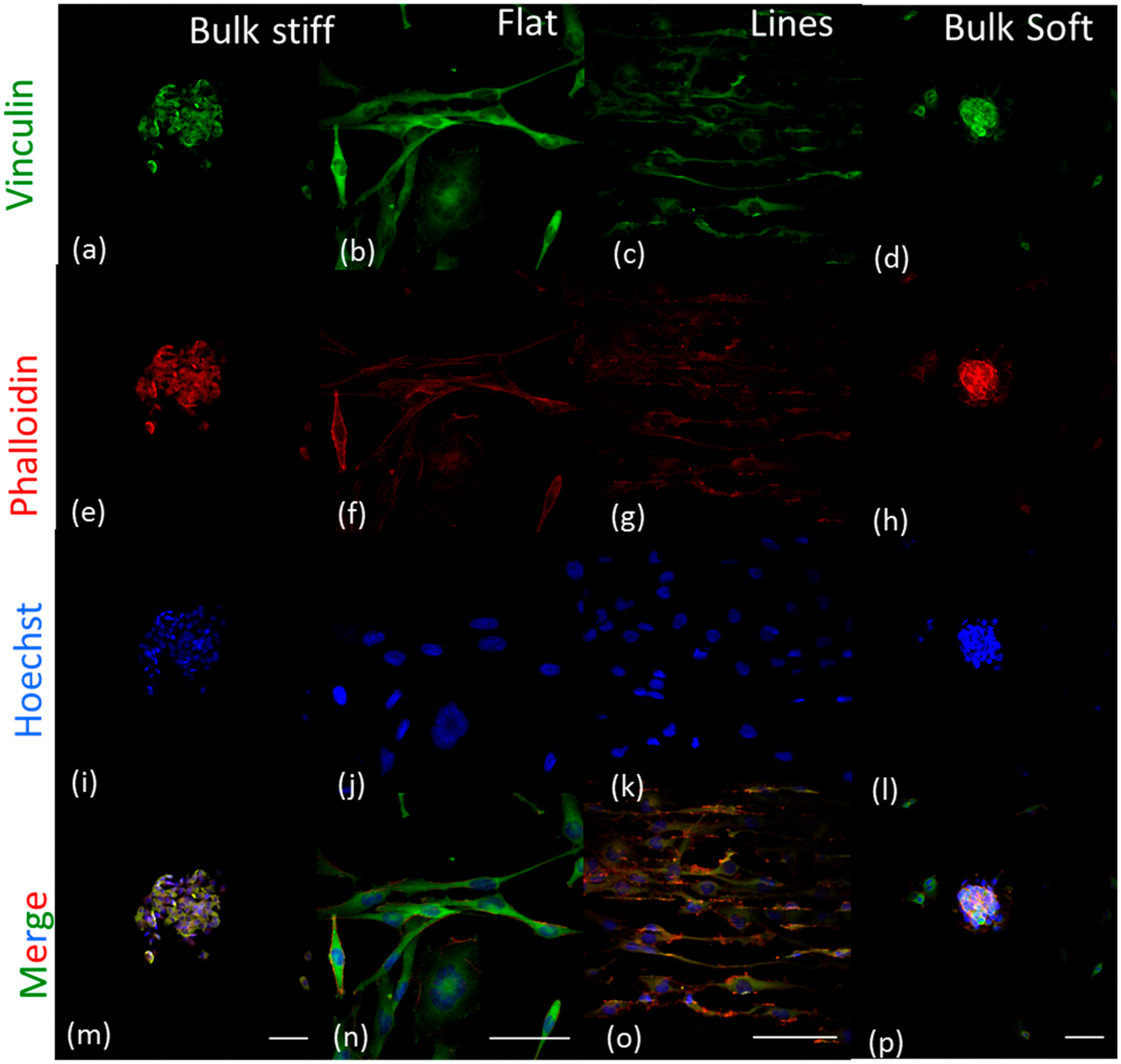

Figure S3: Distinctive mechanical stiffness in the diverse microenvironments is dependent on FAK signaling. Representative fluorescence images of phalloidin-stained F-actin, (red), vinculin for focal adhesion proteins (green) and HOECHST 33258 for cell nuclei (blue) of the GL15 on the different durotactic substrates: bulk very stiff (a,e,i,m), durotactic gradient flat (b,f,j,n), durotactic gradient with grooves (Lines)(c,g,k,o) and bulk very soft (d,h,l,p) . The images are representative of one of three experiments conducted in duplicate. The images m,n,o, p are the merge of the fluorescence GFP (FITC), falloidina rodamina-coniugata, e HOECHST 33258. Scale bar 100µm.

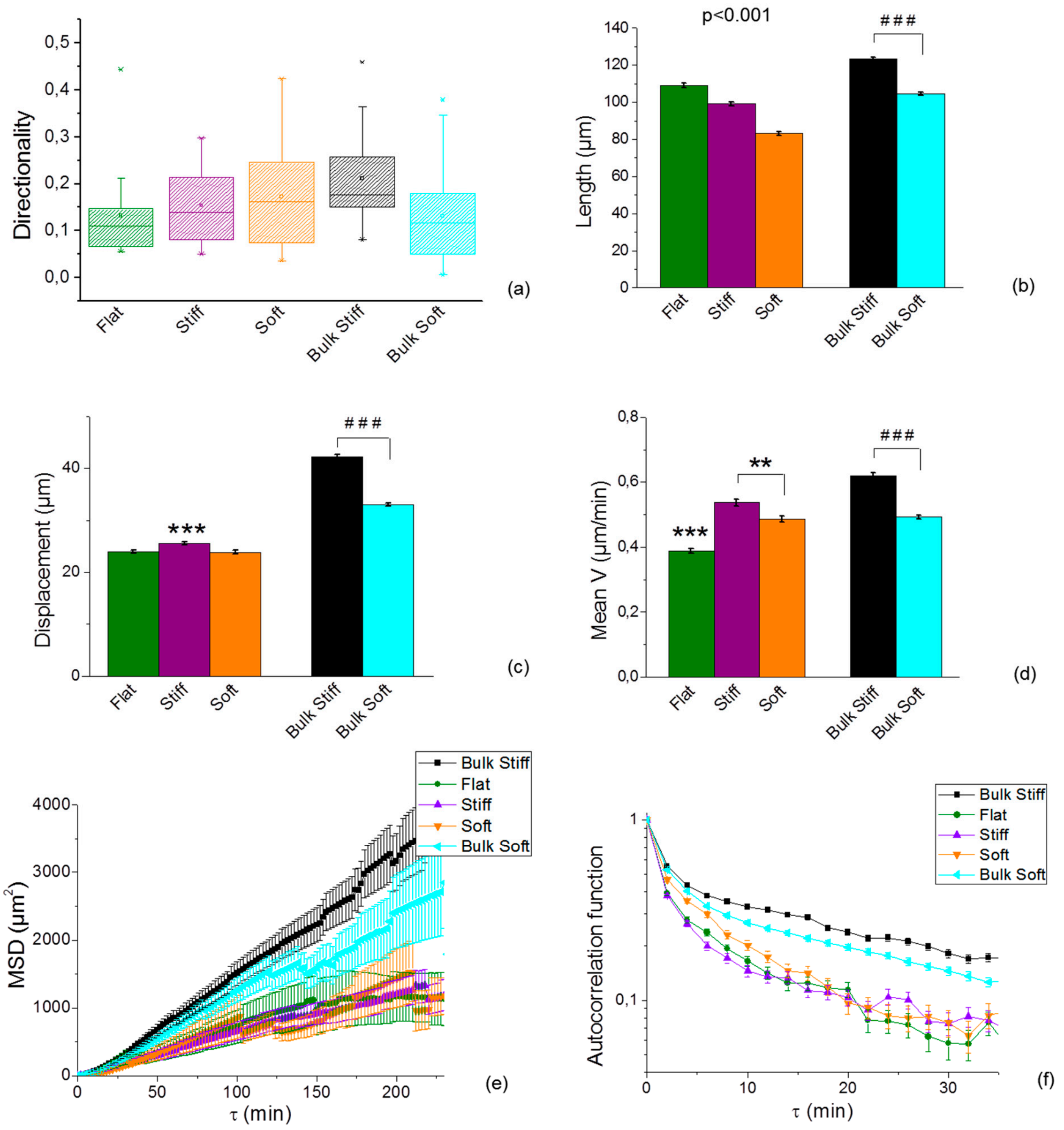

Figure S4: GL15 glioma cells' dynamic properties correlates to the mechanical stiffness of the substrate showing a decreased response on the durotactic substrates. Plots show a) directionality b) net displacement, c) path length, d) average instantaneous speed of migration of cells imaged for over eight hours on the different substrates. e) Plot of MSD vs. time indicating more directed motion for cells on the gradient durotactic micropatterned substrates than for cells on bulk substrates. f) plot of average spatial autocorrelation function showing that on durotactic substrates GL15 cell moves with a more highly correlated velocity than on the bulk substrates. Analyses were performed on tracks pooled from four independent experiments, from 38 cells. Statistical significance indicated by * for p <0.05, ** for p<0.01 and *** for p <0.0001, were assessed by Tukey one-way ANOVA test. The hash tag indicates statistical significance by two-tailed Student's t-test analysis with ### for p <0.0001. Error bars indicate s.e.m.

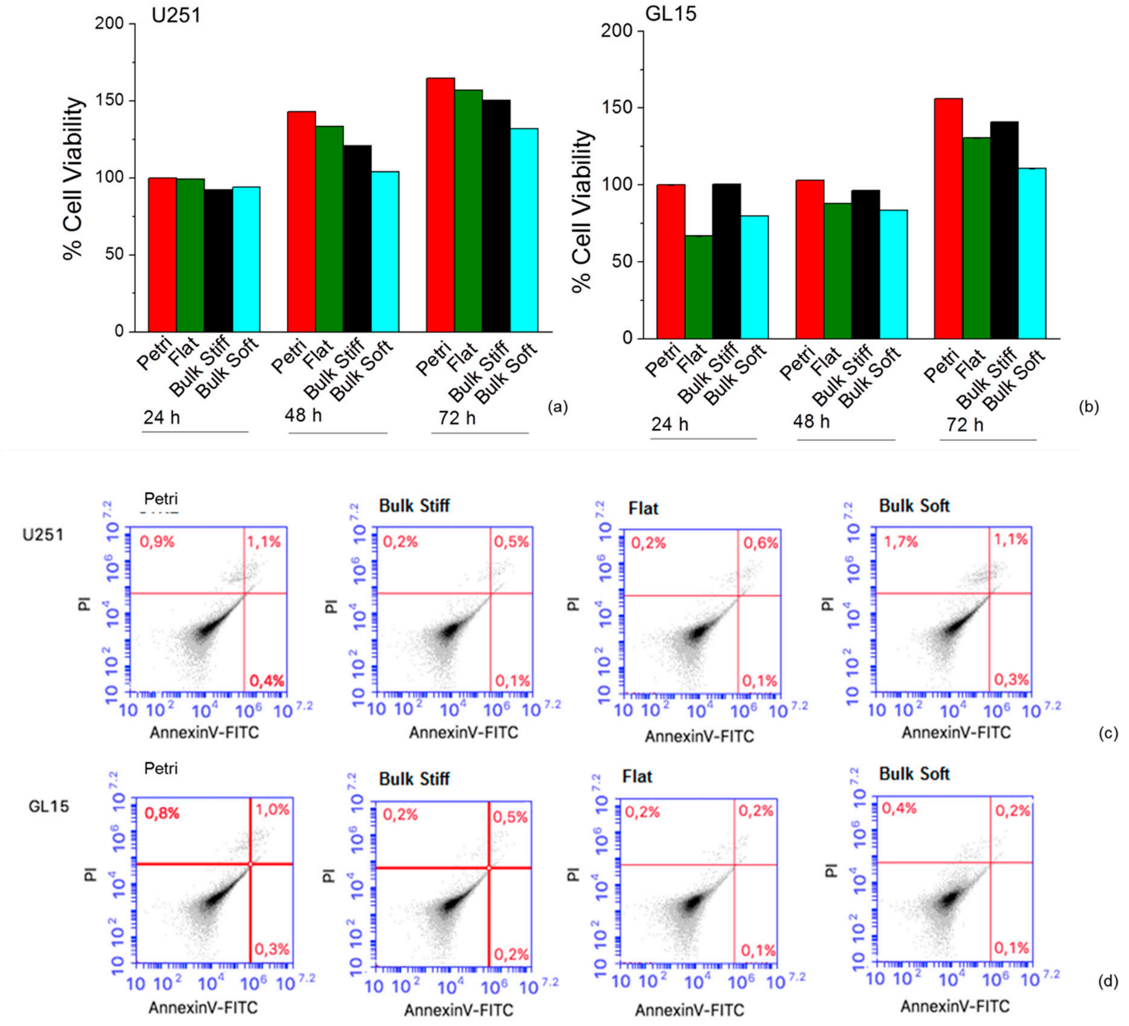

Figure S5: Durotactic flat substrates promotes glioma cell proliferation respect to uniform bulk. Plots on durotactic flat and bulk stiff and soft substrates after 24, 48, 72 h of culture measured by MTT assay of a) U251 and b) GL15 and cell apoptosis measured by flow cytometry using annexin V-FITC/PI double staining on bulk and durotactic flat substrate of c) U251 and d) GL15 cells; the petri dish is used as the control.

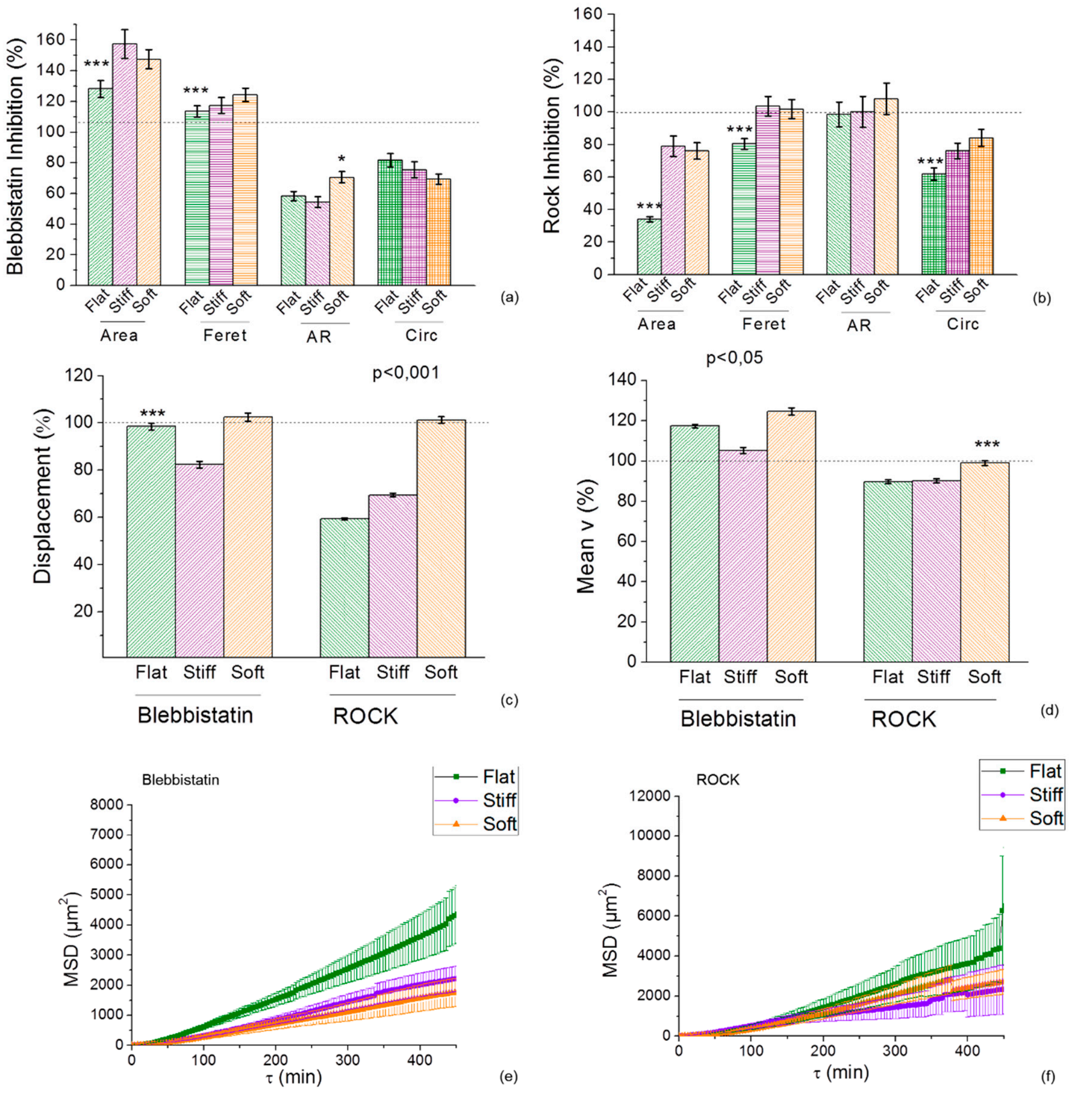

Figure S6: Durotactic substrates altered the responses of GL15 cells to inhibition of NMMII and ROCK Y27632, responsible for cytoskeleton assembly and cell contractility. GL15 cell morphology subsequent inhibition was sensitive to the durotactic micropatterned substrates compared to uniform substrates. Effects of Blebbistatin and ROCK Y27632 on a) cellular spread area, elongation (Feret diameter), A.R. and Circularity for each substrate stiffness. Treated cells (bars) are compared with that in control cells (dotted lines). Relative to the morphologic cell response on uniform substrates, GL15 increased their spread area and elongation on the durotactic gradient substrates with inhibitors. Cell track positions were used to calculate the displacement (c), speed (d) of migration and the mean squared displacement (e, f) with Blebbistatin (25μM, 8 hrs), and ROCK Y27632 (10 μM, 8 hrs). Statistical significance indicated by * for $p < 0.05$, and *** for $p < 0.0001$, assessed by Tukey one-way ANOVA test.

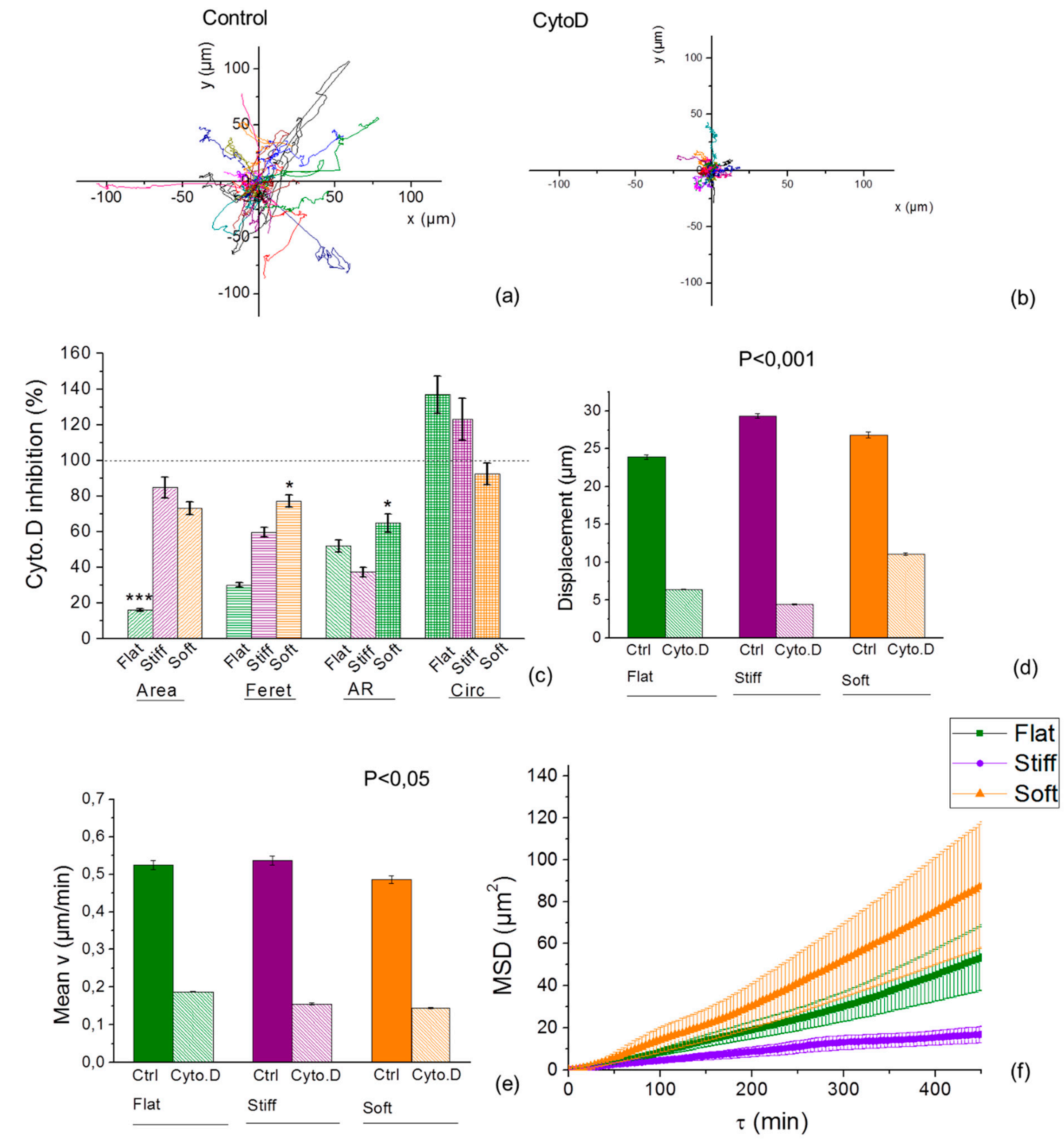

Figure S7: Inhibition of actin polymerization with Cyto. D on GL15 on durotactic substrates showed a stiffness-dependent reduced response with a higher sensitivity to soft substrates. (a-b) Cell movement track of 30 cells from 4 fields of acquisition on flat and durotactic substrates analysed by ImageJ. The different colours represent different cells analysed. c) GL15 cell morphology such as cellular spread area, elongation (Feret diameter), A.R. and Circularity of cells subsequent treatment with Cyto D was sensitive to the gradient stiffness compared to durotactic uniform substrates. Cell track positions were used to calculate the displacement (d), speed of migration (e) and the mean squared displacement (MSD) (f) as a function of the time lag (τ) of of GL15 cells. Statistical significance indicated by * for p <0.05, assessed by Tukey one-way ANOVA test.

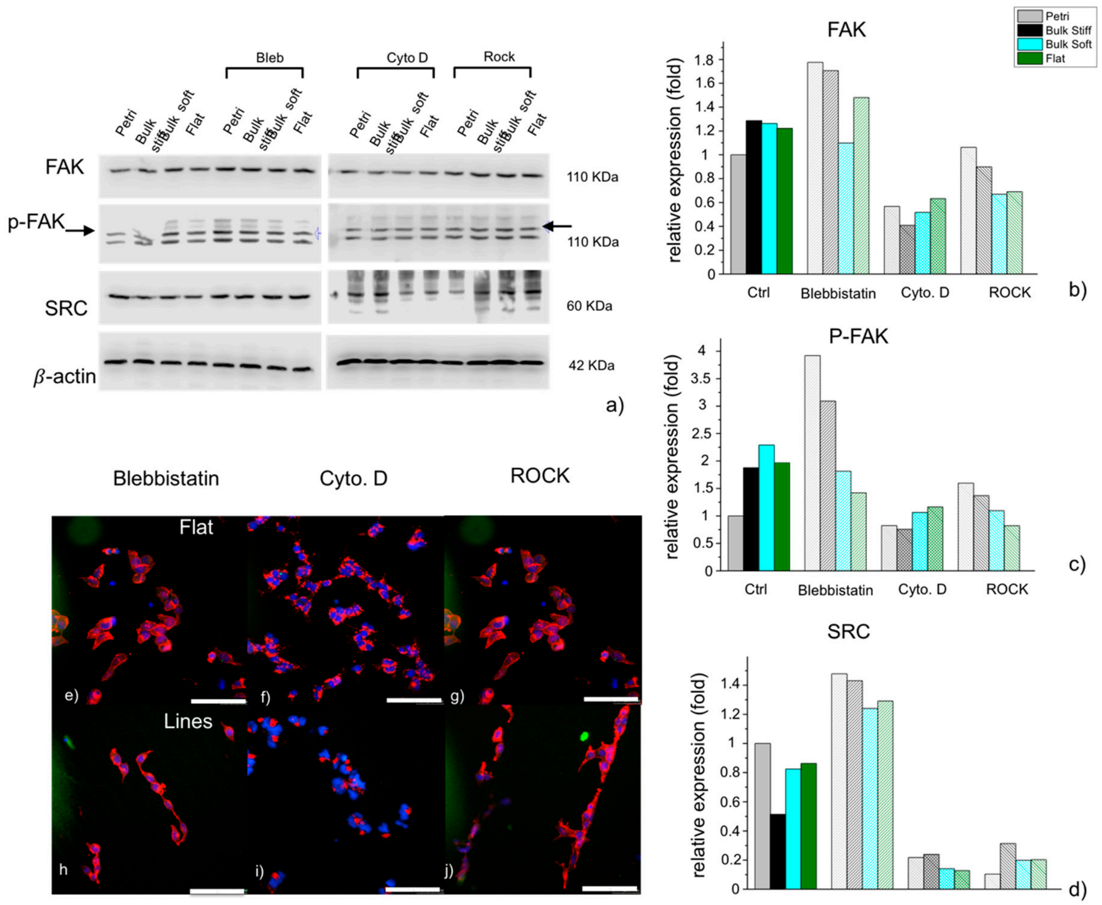

Figure S8: FAK, Phosphorylation of FAK Tyr397 and SRC in glioma cells depends on substrate stiffness. Western blot analysis (a) and densitometry of b) FAK, c) phosphorylation of FAK Ty^{r397} and d) SRC levels in lysates from GL15 cells cultured on bulk stiff, bulk soft and flat durotactic , the Ctrl is the Petri dish. Quantified values shown in the graph ($n \geq 3$). FAK, P-FAK and Src expression levels were normalized to the β-actin. Immunofluorescence staining merge of phalloidin, vinculin and HOESCHT showing actin cytoskeleton organization of GL15 cells treated with inhibitors on flat substrates with e) Blebbistatin f) ROCK Y-27632, and g) Cyto D and micropatterned durotactic lines with h) Blebbistatin i) ROCK Y-27632, and j) Cyto. D. Scale bar 100 μm.
